# Protective effects of cannabidiol against nephrotoxicity of ionophore coccidiostats in HEK-293 cells

**DOI:** 10.2478/jvetres-2026-0047

**Published:** 2026-08-24

**Authors:** Oliwia Maria Kończak, Joanna Zeyland, Lidia Radko

**Affiliations:** 1 Department of Biochemistry and Biotechnology, Poznań University of Life Sciences, 60-632 Poznań, Poland; 2 Department of Preclinical Sciences and Infectious Diseases, Faculty of Veterinary Medicine and Animal Sciences, Poznań University of Life Sciences, 60-637 Poznań, Poland

**Keywords:** cannabidiol, coccidiostats, nephrotoxicity, HEK-293

## Abstract

**Introduction:**

Veterinary drug residues in the environment threaten public health and animal welfare. Ionophore coccidiostats (lasalocid (LAS), monensin (MON) and narasin (NAR)) are commonly used in animal production. Overdosing, errors in feed mixing or interactions with other drugs can cause nephrotoxicity in animals, which has also been observed in poisoned humans. The toxic effects of veterinary drugs may be mitigated by cannabidiol (CBD), known to be antioxidant and cytoprotective and expected to have a protective effect on kidney cells. The aim of this study was to evaluate the effect of CBD on cytotoxicity induced by ionophore coccidiostats in human embryonic kidney cells (HEK-293).

**Material and Methods:**

Cells were exposed to LAS, MON and NAR, either alone or in combination with CBD. Cytotoxicity was assessed using mitochondrial activity, lysosomal activity, total protein content and membrane integrity assays. The action of combination of CBD and the coccidiostats was determined using interaction analysis.

**Results:**

The ionophores induced cytotoxicity dose-dependently. Lasalocid was the most toxic, significantly impairing mitochondrial function and membrane integrity at 1 µg/mL. Co-treatment with CBD significantly attenuated the drugs’ toxic effects. Predominantly, antagonistic effects were revealed, indicating a protective effect of CBD. However, an additive effect was observed at high concentrations of the LAS-CBD combination.

**Conclusion:**

Cannabidiol exerts a protective effect against coccidiostat nephrotoxicity in human renal cells. This finding suggests its modulation of cellular responses to ionophore-induced cytotoxicity, offering a basis for further *in vivo* studies on its potential.

## Introduction

The presence of veterinary drugs in the environment and food chain poses a serious challenge to One Health. Ionophore coccidiostats such as lasalocid, monensin and narasin are veterinary drugs which are widely used in animal production to prevent and control coccidiosis (11). Errors made during coccidiostat therapy can lead to residues in edible animal tissues and eggs (1, 2, 18, 19). Accidental consumption of feed or food contaminated with these residues has poisoned both animals and humans, sometimes fatally (3, 5, 17, 26, 31). The clinical symptoms of poisoning included multi-organ dysfunction, including renal dysfunction, leading to metabolic and excretory disorders (3, 8, 17, 26, 31). This toxicity is associated with the disruption by ionophores of the transport of monovalent and divalent cations across biological membranes, thereby perturbing ion homeostasis and impairing cellular and energy metabolism, ultimately leading to cell death (7, 11, 24).

Literature data indicate that the kidneys are particularly susceptible to toxicity caused by coccidiostats because of their role in the filtration, metabolism and excretion of these drugs (23). Chronic exposure to low concentrations of coccidiostats present in eggs may pose a risk to human health, highlighting the need for toxicological studies using *in vitro* models. The human embryonic kidney cell line HEK-293 is widely used in nephrotoxicity studies and is a suitable cell model for assessing cytotoxic responses at the cellular level (21, 23, 25).

The cytotoxic effects of ionophore coccidiostats can be partly attenuated by plant-derived substances (known as phytobiotics). Recent studies have demonstrated the protective effect of cannabidiol (CBD) on human cells after exposure to drugs such as tiamulin and doxycycline (21, 25). Cannabidiol is a non-psychoactive phytocannabinoid derived from *Cannabis sativa* L. that has gained attention because of its anti-inflammatory, antioxidant and cytoprotective properties (13, 20, 21, 25, 28, 29). These properties suggest that CBD may modulate cellular responses to xenobiotics, including veterinary drugs. Given the increasing availability of CBD-containing products and scientific evidence suggesting CBD’s protective effect against drug-induced cytotoxicity, it is important to evaluate potential interactions between CBD and ionophore coccidiostats. The aim of this study was to evaluate the effect of CBD on the cytotoxicity induced by lasalocid, monensin and narasin in HEK-293 cells by investigating mitochondrial activity, lysosomal function, cell proliferation and cell membrane integrity. Additionally, based on the endpoints studied, the nature of the interactions between the veterinary drugs studied and CBD was assessed.

## Material and Methods

**Drugs and chemical reagents.** An analytical standard of cannabidiol (CBD; CAS 13956-29-1, certified absolute purity) was obtained from PhytoLab (Vestenbergsgreuth, Germany). Coccidiostats, namely lasalocid sodium salt (LAS; CAS 25999-31-9, >90%), monensin sodium salt hydrate (MON; CAS 22373-78-0, 90–95%) and narasin sodium salt (NAR; CAS 55134-13-9, 98%) were purchased from Sigma-Aldrich (St. Louis, MO, USA). The reagents needed for cell culture and testing were Triton X-100, trypan blue, dimethyl sulphoxide (DMSO), FBS, neutral red (NR), Coomassie Brilliant Blue R-250, 3-[4,5-dimethylthiazol-2-yl]-2,5-diphenyltetrazolium bromide (MTT), trypsin–EDTA, L-glutamine and antibiotic solution (10,000 U/mL penicillin and 10 mg/mL streptomycin), were also purchased from Sigma-Aldrich. Roche Diagnostics (Mannheim, Germany) supplied the Cytotoxicity Detection LDH kit. All other reagents were purchased from commercial suppliers and were of the highest available purity.

**Cell cultures.** The study used HEK-293 human embryonic kidney cells (CRL-1573), which were purchased from the American Type Culture Collection. Cells were cultured in MEM (Sigma-Aldrich) with 10% FBS, 1% L-glutamine and 1% antibiotic at 37°C in 5% CO₂ in 75 cm² flasks (Nunc, Roskilde, Denmark). The MEM was changed every 2–3 days. Cells were passaged at 70–80% confluence using 0.25% trypsin–0.02% EDTA. After passaging, single-cell suspensions (1 × 10⁵^5^cells/mL) were seeded into 96-well plates at 100 µL/well and pre-incubated for 24 h before exposure to the ionophore coccidiostats or coccidiostat CBD mixtures.

**Exposure of cells to substances.** Stock solutions of LAS, MON, NAR and CBD were prepared in DMSO. Working solutions of coccidiostats at 1, 5, 10 and 50 µg/mL and of CBD at 1.56 µg/mL were made in antibiotic-free medium with 1% FBS. Test concentrations of substances were chosen as per the literature (7, 21, 25) and pilot test outcomes. Final concentrations of DMSO were 0.1% for single drugs or 0.2% for mixtures. Cells were treated with 0.1% or 0.2% DMSO as negative controls, and 1% Triton X-100 was used as the positive control. The viability/cytotoxicity was assessed after 72 h of exposure of the cells to the drugs or their mixtures with CBD. The medium was not changed during the incubation time. The viability/cytotoxicity was assessed using the four assays described below.

**MTT assay.** It relies on the ability of viable cells to convert tetrazolium dye into an intracellular dark formazan through the activity of mitochondrial reduced nicotinamide adenine dinucleotide phosphate—dependent dehydrogenases. The quantity of the resulting formazan correlates with the number of metabolically active cells and is subsequently measured using colorimetric detection. After exposure, 10 µL of MTT (5 mg/mL in PBS) was added per well and incubated for 3 h. The dye was removed, the formazan was dissolved in 100 µL DMSO, and the plates were shaken for 15 min at room temperature. Absorbance was read at 570 nm in a Synergy 2 Multi-Mode Microplate Reader (BioTek Instruments, Winooski, VT, USA) and referenced to a blank. Cytotoxicity was expressed as % of the negative control (25).

**Neutral red uptake assay.** It is based on staining viable cells with NR, a dye that diffuses via the plasma membrane and accumulates in lysosomes. Viable cells retain the dye but damaged or dead cells cannot. After incubation, the treatment medium was removed and the cells were rinsed with PBS. Then 100 µL of NR (50 µg/mL) was added per well, the plates were incubated for 3 h, the cells were washed with PBS and dye was extracted with acetic acid : ethanol : water (1 : 50 : 49, v/v/v). After shaking for 10 min, absorbance was read at 540 nm in the Synergy 2 and referenced to a blank. Cytotoxicity was expressed as % of the negative control (25).

**Total protein content (TPC) assay.** It is based upon staining cellular protein. After the incubation, medium containing the test compounds was removed and 100 µL of Coomassie Brilliant Blue R-250 solution was added to each well. The plate was shaken for 10 min. The stain was removed and the cells were rinsed twice with 150 µL of washing solution, which was acetic acid : ethanol : water (5 : 10 : 85, v/v/v). Next, 100 µL of 1 M potassium acetate was added and the plates were shaken for 10 min to desorb. Absorbance was read at 595 nm in the Synergy 2 and referenced to a blank. Cytotoxicity was expressed as % of the negative control (25).

**Lactate dehydrogenase assay** It relies on evaluating cell membrane injury by quantifying LDH released into the extracellular medium. The assay was performed according to the manufacturer’s protocol. Aliquots of 100 µL of the medium without cells were transferred into the wells of a transparent, flat-bottomed 96-well plate, after which 100 µL of the reaction mixture was added to each well. The plates were incubated for 30 min at room temperature in the dark. After incubation, 50 µL of 1 M HCl was added to each well to stop the reaction. Absorbance was measured at 492 nm using the Synergy 2, with a blank used as the reference (25).

**Assessment of synergistic/antagonistic effects.** The method established by Chou and Talalay ([Bibr j_jvetres-2026-0047_ref_006]) was used to determine the types of interaction that occur when cells are exposed to combinations of coccidiostats and CBD. The dose–effect relationships of the individual and combined test compounds were modelled biometrically using the median-effect equation based on the law of mass action:


$${\text{F}}_{\text{a}}/{\text{F}}_{\text{u}}={\left(\text{D}/{\text{D}}_{\text{m}}\right)}^{\text{m}}$$

where D is the dose of veterinary drugs or CBD, Fa is the fraction affected by D, Fu is the fraction unaffected (*i.e*. Fu = 1 − Fa), Dm is the median-effect dose (e.g. half maximal inhibitory concentration) and m is the coefficient signifying the shape of the dose–effect relationship. When the coefficient m was 1, >1 and <1 it indicated hyperbolic, sigmoidal and flat sigmoidal dose—effect curves, respectively.

In this isobolographic analysis, the combination index (CI) is a quantitative parameter used to evaluate the type and strength of interactions between multiple compounds. For all combinations, CI values were calculated across a range of affected fractions from 0.05 to 0.95 (corresponding to 5–95% toxicity). Combination index values of 0.9–1.1, <0.9 and >1.1 indicated additive, synergistic and antagonistic effects, respectively.

**Statistical analysis.** The obtained data were presented as mean values $\overset{-}{x}$ with SD. Cytotoxicity results were analysed by one way ANOVA, followed by Dunnett’s test. The IC₅₀ (half maximal inhibitory concentration – the concentration of the drug causing a 50% decrease in cell viability compared to the negative control regarded as 100%) was calculated using GraphPad Prism 5.0 (GraphPad Software, Boston, MA, USA). To compare IC₅₀ values statistically between groups, ANOVA with Tukey’s *post-hoc* test was applied. Differences were considered statistically significant at P-value ≤ 0.05.

## Results

Treatment of HEK-293 cells with LAS caused mitochondrial dysfunction and cell membrane destabilisation at a concentration of 1 µg/mL (Fig. 1), while inhibition of cell proliferation and lysosomal activity was observed at a concentration of 5 µg/mL. Co-treatment with CBD mitigated these cytotoxic effects, resulting in increased cell viability in all assays (Fig. 1). While IC₅₀ values in the MTT assay remained unchanged after CBD supplementation, these values were almost twofold higher in the TPC assay and fourfold higher in the LDH assay for the LAS-CBD combination compared with the IC₅₀ value for LAS alone (Tables 1). Interaction analysis in the MTT assay showed clear antagonism between CBD and the drug at low concentrations. In contrast, an additive effect was observed at high concentrations of LAS and CBD. Clear antagonism between CBD and LAS, regardless of drug concentration, was observed in the other tests, suggesting that CBD has a protective effect against LAS-induced nephrotoxicity in human kidney cells (Fig. 1).

**Fig. 1.**
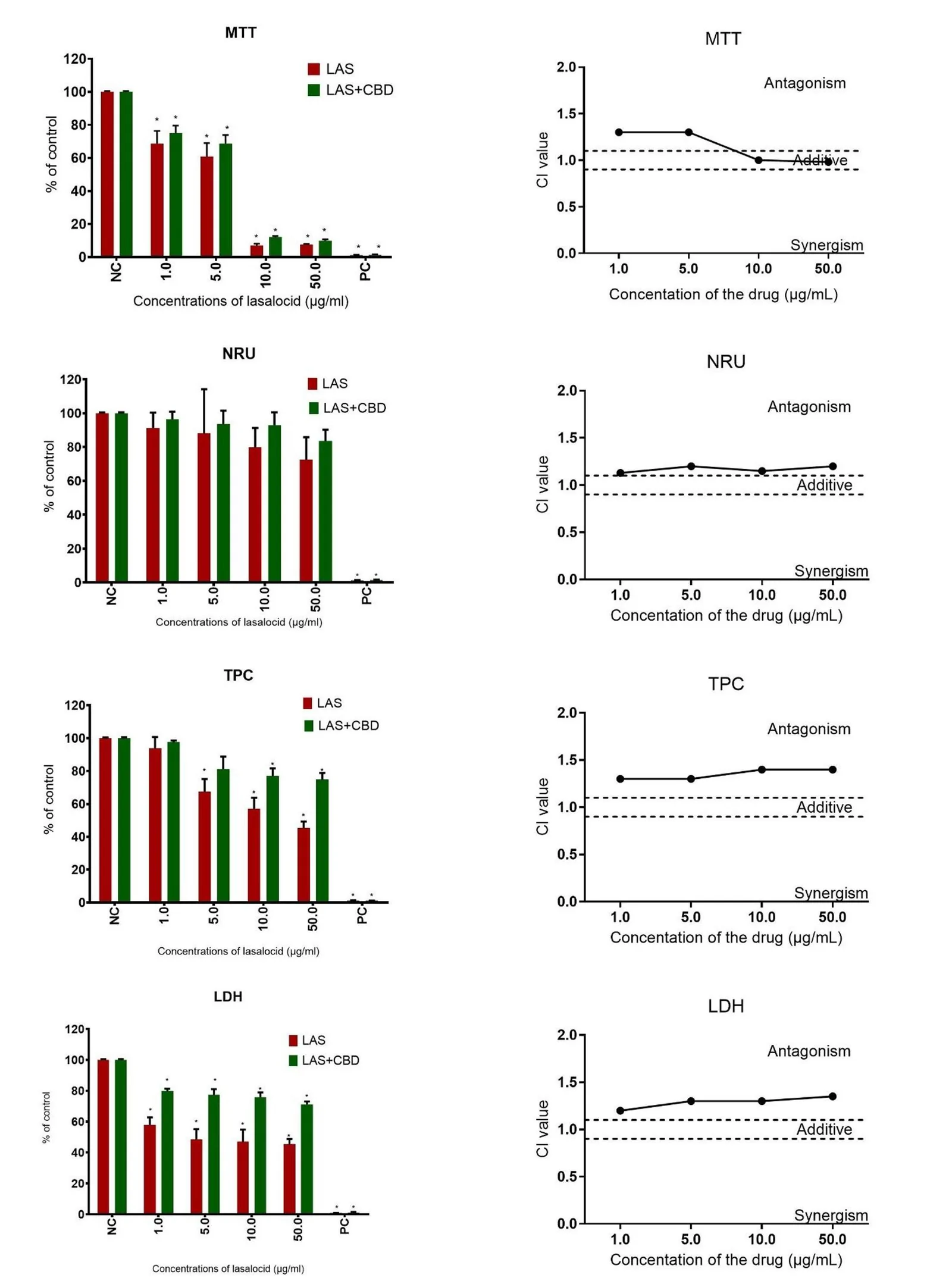
Effects of the joint action of lasalocid (LAS) and cannabidiol (CBD) on human embryonic kidney HEK-293 cell viability. The cytotoxicity and character of interaction was assessed using 3-[4,5-dimethylthiazol-2-yl]-2,5-diphenyltetrazolium bromide (MTT), neutral red uptake (NRU), total protein content (TPC) and lactate dehydrogenase (LDH) assays following a 72-h exposure period. Vertical bars represent 95% confidence intervals for the combination index (CI) values, calculated via sequential deletion analysis. The horizontal dashed line (CI = 0.9–1.1) indicates the additive threshold, where values below the line signify synergism and values above signify antagonism. Data are expressed as mean ± SD from three independent experiments (n = 3). NC – negative control; PC – positive control; * – statistical significance at P-value ≤ 0.05

Treatment of HEK-293 cells with MON at a concentration of 10 µg/mL resulted in a decrease in lysosomal activity, while the highest concentration tested of 50 µg/mL led to inhibition of mitochondrial activity and proliferation, as well as destabilisation of the cell membrane (Fig. 2). Simultaneous administration of CBD with MON mitigated these cytotoxic effects, resulting in increased cell viability in all tests (Fig. 2). The IC₅₀ values for MON after 72 h of exposure of kidney cells were higher than 50 µg/mL (Tables 1). Analysis of the nature of the interaction between CBD and MON revealed antagonism, indicating a protective effect of CBD against the drug’s nephrotoxic action (Fig. 2).

**Fig. 2.**
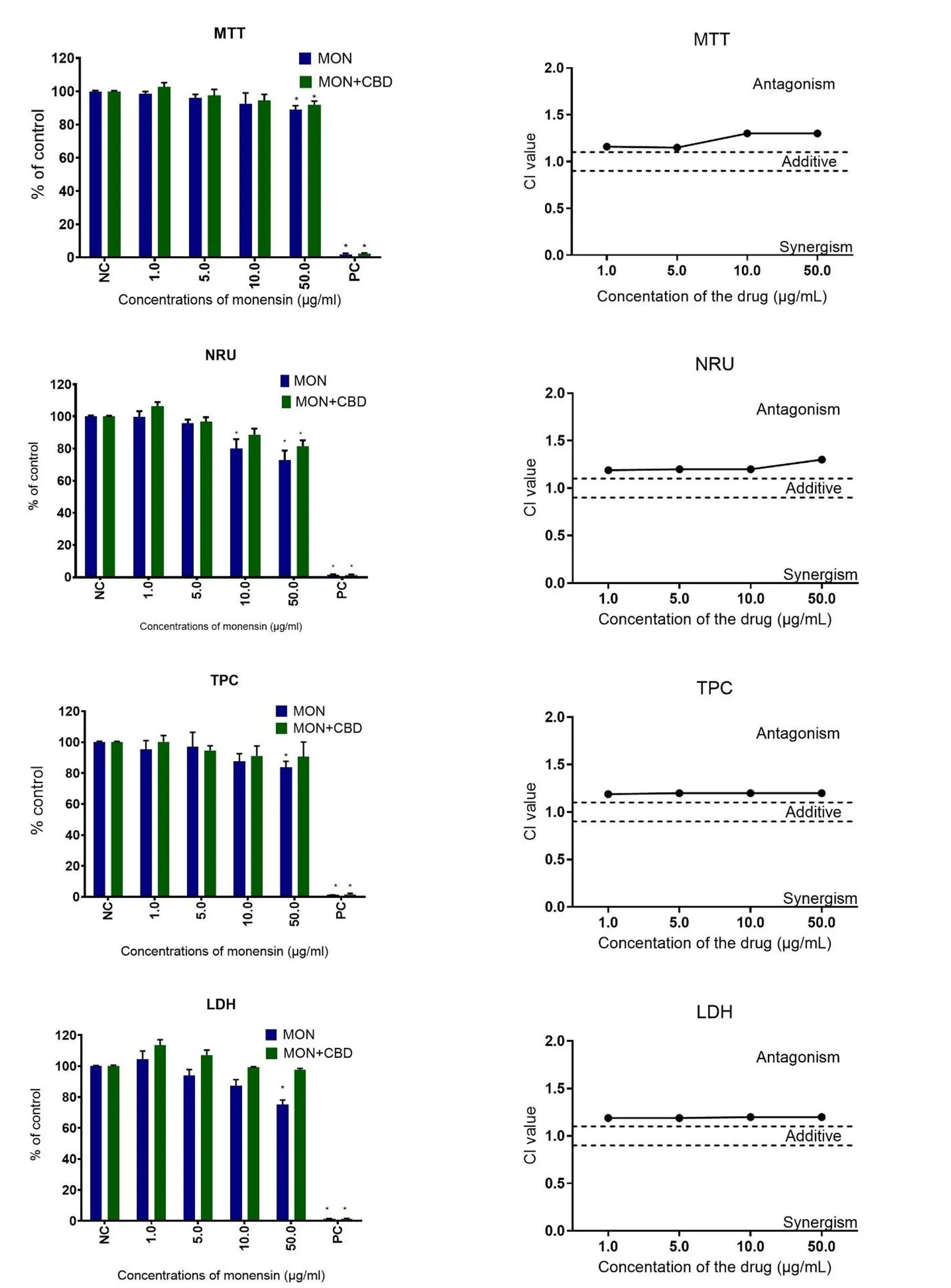
Effects of the joint action of monensin (MON) and cannabidiol (CBD) on human embryonic kidney HEK-293 cell viability. The cytotoxicity and character of interaction was assessed using 3-[4,5-dimethylthiazol-2-yl]-2,5-diphenyltetrazolium bromide (MTT), neutral red uptake (NRU), total protein content (TPC) and lactate dehydrogenase (LDH) assays following a 72-h exposure period. Vertical bars represent 95% confidence intervals for the combination index (CI) values, calculated via sequential deletion analysis. The horizontal dashed line (CI = 0.9–1.1) indicates the additive threshold, where values below the line signify synergism and values above signify antagonism. Data are expressed as mean ± SD from three independent experiments (n = 3). NC – negative control; PC – positive control; * – statistical significance at P-value ≤ 0.05

**Table 1.** Half-maximal inhibitory concentration values (µg/mL) calculated in human embryonic kidney HEK-293 cell cultures after 72-h exposure to lasalocid (LAS), monensin (MON), narasin (NAR) and their mixtures with cannabidiol (CBD)

Method	LAS	LAS + CBD	MON	MON + CBD	NAR	NAR + CBD
MTT	8.0 ± 1.6	8.4 ± 0.3	> 50	> 50	5.7 ± 0.4	45.6 ± 9.5
NRU	> 50	> 50	> 50	> 50	> 50	> 50
TPC	27.9 ± 10.5	> 50	> 50	> 50	> 50	> 50
LDH	12.3 ± 2.8	> 50	> 50	> 50	> 50	> 50

MTT – 3-[4,5-dimethylthiazol-2-yl]-2,5-diphenyltetrazolium bromide assay; NRU – neutral red uptake assay; TPC – total protein content assay; LDH – lactate dehydrogenase assay. Data are presented as mean ± SEM from three independent experiments (n = 3)

Exposure of HEK-293 cells to narasin (NAR) caused a concentration-dependent decrease in mitochondrial activity. After exposure to the highest concentration tested (50 µg/mL), cell proliferation inhibition and cell membrane destabilisation were observed. No significant changes in lysosomal activity were observed (Fig. 3). Co-administration of CBD with NAR mitigated the cytotoxic effects caused by the veterinary drug, resulting in increased cell viability in all tests. In the MTT assay, the IC₅₀ value for the CBD-NAR mixture increased almost eightfold compared to the value for the drug alone (Tables 1). Analysis of the nature of the interaction between CBD and the veterinary drug revealed antagonism, indicating a protective effect of CBD against narasin-induced nephrotoxicity (Fig. 3).

**Fig. 3.**
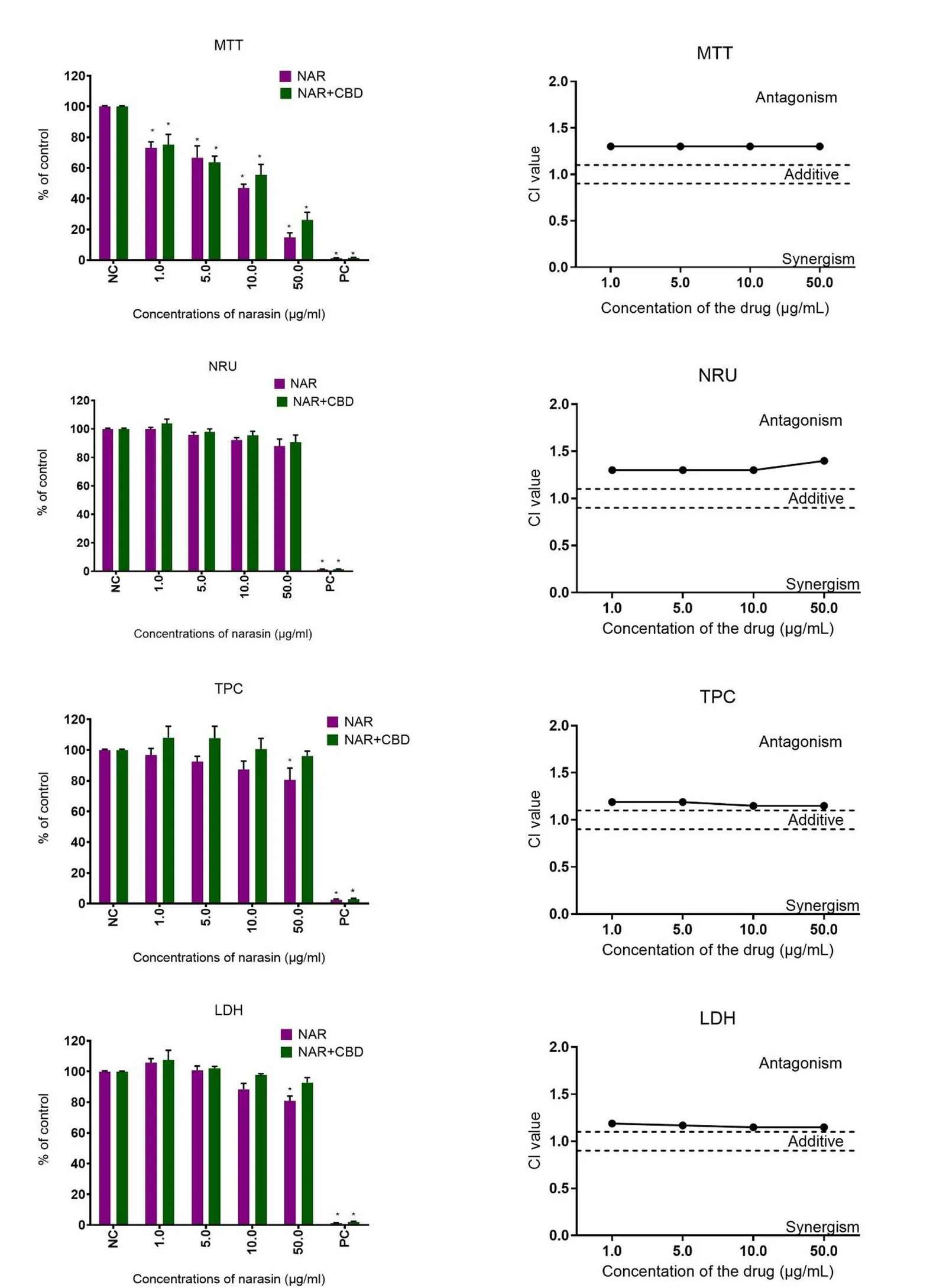
Effects of the joint action of narasin (NAR) and cannabidiol (CBD) on human embryonic kidney HEK-293 cell viability. The cytotoxicity and character of interaction was assessed using 3-[4,5-dimethylthiazol-2-yl]-2,5-diphenyltetrazolium bromide (MTT), neutral red uptake (NRU), total protein content (TPC) and lactate dehydrogenase (LDH) assays following a 72-h exposure period. Vertical bars represent 95% confidence intervals for the combination index (CI) values, calculated via sequential deletion analysis. The horizontal dashed line (CI = 0.9–1.1) indicates the additive threshold, where values below the line signify synergism and values above signify antagonism. Data are expressed as mean ± SD from three independent experiments (n = 3). NC – negative control; PC – positive control; * – statistical significance at P-value ≤ 0.05

## Discussion

The presence of ionophore coccidiostats in the environment and food chain poses a serious threat to both human and animal health. Renal impairment is one of the most severe clinical manifestations of poisoning with ionophore coccidiostats in both humans (3, 17, 31) and animals (4, 8, 10, 12). In camels and turkeys intoxicated by these drugs, gross lesions included pale enlarged kidneys, and dark brown urine was observed in the urinary bladder. Histopathological findings included twisted renal tubules, which also showed varying degrees of degeneration and necrosis and focal haemorrhages (8, 12). A case report in horses described renal histopathological lesions including congestion, haemorrhage, calcification and degeneration of the renal tubules (9). In dogs poisoned with MON, renal medullary necrosis was observed (4). Monensin intoxication in goats led to a wide range of biochemical abnormalities, and the evident renal damage was congestion and necrosis of the renal tubules (10). Increased serum creatinine concentrations were observed in the goat study and may have reflected renal dysfunction; they are considered a common biochemical abnormality associated with MON poisoning in animals. In human poisoning, rhabdomyolysis may result in acute renal failure, which represents the main life-threatening complication (5, 17, 31).

*In vitro* studies have confirmed the nephrotoxic effect of coccidiostats, particularly of monensin (15). Studies showed that MON acted antiproliferatively by activating autophagy and reducing the level of the papillary renal cell carcinoma–transcription factor E3 fusion transcript in the UOK146 renal cell line (30). The drug inhibited the growth of renal cell carcinoma cells (IC₅₀ = 2.5 μM) by arresting the cell cycle or apoptosis (22). Monensin was shown to reduce the rate of recovery of V2 vasopressin receptors to the cell membrane, as well as the dissociation (removal) of the hormone from LLC-PK1 (Lilly Laboratories culture porcine kidney 1) renal epithelial cells (14). This coccidiostat was also shown to cause marked ultrastructural changes in proximal tubular cells in the renal cortex of piglets (27). This study demonstrated not only that MON exerted a significant cytotoxic effect on human kidney cells, but that LAS and NAR also did, and that these effects could be effectively modulated by cannabidiol.

Given the widespread use of ionophores in livestock farming and the potential risk of accidental human exposure through the food chain, understanding the mechanism of nephrotoxicity and finding potential cytoprotective agents is of great clinical importance. Our results indicated that all three coccidiostats tested induced dose-dependent cytotoxicity, manifested primarily by mitochondrial dysfunction, cell membrane destabilisation and inhibition of cell proliferation. This is consistent with the established mechanism of action of ionophores, which act as mobile cation carriers, disrupting intracellular ion gradients and causing mitochondrial ion overload and oxidative stress (16, 24). The HEK-293 kidney cell model proved to be particularly sensitive to LAS, with toxicity observed at concentrations as low as 1 µg/mL, while MON and NAR required higher concentrations (10–50 µg/mL) to cause significant damage. This suggests that although the overall mechanism of action is common, the potency and kinetics of renal damage could differ depending on the specific ionophore.

The most important result of this study is the significant reduction in toxicity caused by coccidiostats after simultaneous administration of CBD. Analysis of interactions consistently showed antagonism, which in toxicological terms indicates a protective effect. Interestingly, in the case of NAR, CBD supplementation attenuated the loss of mitochondrial activity of HEK-293 cells caused by the ionophore, preserving it at an almost eightfold higher level than that observed after treatment with the drug alone. This cytoprotective effect of CBD is likely due to its multifaceted pharmacological profile. Unlike classic antioxidants such as silybin, which primarily stabilise cell membranes (7, 24), CBD acts through several pathways. The antioxidant activity of CBD is known to reduce the production of reactive oxygen species, which are a major factor in ionophore induced apoptosis (16, 20). By modulating mitochondrial homeostasis, CBD may prevent the ‘metabolic collapse’ typically observed during ionophore poisoning. Through receptor-mediated signalling, CBD's interaction with transient receptor potential vanilloid (TRPV) channels and cannabinoid receptors may further stabilise cellular responses to ionic imbalances (28). Our results are consistent with previous reports on phytobiotics, *e.g*. those by Cybulski *et al*. (7) and Radko *et al*. (24), who demonstrated that silybin reduces the toxicity of coccidiostats in liver (HepG2) and muscle (L6) cells. However, the present study extends these findings to a human kidney model, which is particularly relevant as the kidneys are the main site of xenobiotic excretion and are highly susceptible to drug-induced damage. Interestingly, although the interaction was mainly antagonistic (protective), the LAS-CBD combination showed an additive effect at high concentrations. This suggests that the protective capacity of CBD may have a threshold, or that at very high concentrations, the combined metabolic burden on mitochondria may exceed the compensatory mechanisms. This highlights the importance of biological context and the concentration-dependent nature of CBD interactions. Elucidating the mechanism underlying the additive effect of both compounds is challenging and requires detailed investigation. Certainly, the pharmacological aspects of both agents and their concentration ratio must be taken into account. Altering this ratio may shift the interaction toward synergism or antagonism. Lasalocid acts as an ionophore, transporting primarily divalent cations, such as Ca^2+^ and Mg^2+^, but also monovalent ones, across cell membranes, which leads to osmotic stress. In turn, CBD potently modulates ion channels (*e.g*. TRPV1 and transient receptor potential melastatin 8) and intracellular calcium stores. The overlap of these two mechanisms may lead to a cumulative influx of calcium into the cytosol, thereby inducing a stronger effect.

## Conclusion

This study provides preliminary evidence that CBD may exert protective effects against LAS-, MON- and NAR-induced nephrotoxicity in human cells. The consistent antagonism observed at the cellular level suggests that CBD could warrant further investigation as an adjunct to mitigate the effects of accidental exposure to ionophores. Further studies, including in vivo investigations, are needed to confirm whether these protective mechanisms translate into systemic detoxification and improved renal clearance.
